# Supplementary material for: Knowledge, attitude and practices of community pharmacists regarding COVID-19: A paper-based survey in Vietnam
Source: PLoS One. 2021 Jul 29;16(7):e0255420. doi: 10.1371/journal.pone.0255420 (PMC8321352; doi:10.1371/journal.pone.0255420)
Supplement: S1 Questionnaire — (DOCX) [file pone.0255420.s001.docx]

**S1 Questionnaire for the survey about KAP of pharmacists regarding COVID-19 in Vietnam**

**............................................................................................................**

| **HANOI UNIVERSITY OF PHARMACY** | Code: ...................................  Day of data collection: ................................... |
| --- | --- |

**QUESTIONNAIRE: KNOWLEDGE, ATTITUDE, AND PRACTICES (KAP) OF**

**COMMUNITY PHARMACISTS REGARDING COVID-19 PANDEMIC IN VIETNAM**

Dear pharmacists. The research team from Hanoi University of Pharmacy is surveying the knowledge, attitude, and practices of community pharmacists regarding the COVID-19 pandemic in Vietnam. The study’s objective is to assess the roles of Vietnamese community pharmacists in activities involving COVID-19 prevention. We truly appreciate your contribution and participation in our research. The time for answering questions is about 10 to 15 minutes. All information in this data collection form is only used for studying. The identity of drugstores and pharmacists will be kept confidential.

| **No.** | **Questions** | **Responses** |
| --- | --- | --- |
| **A. Pharmacists’ profile** | | |
| A1. | What is your gender? | **** Male **** Female |
| A2. | How old are you? |  |
| A3. | What is your highest education attainment? | **** University or higher  **** College  **** Middle |
| A4. | Where is your current residence location? | (Name of province) |
| A5. | How long have you worked in drugstores? (years) |  |
| **B. Knowlege** (Bold responses are right answers) | | |
|  | K1. Ways of COVID-19 transmission (B1-B3): |  |
| B1 | The virus that causes COVID-19 spreads primarily through droplets generated when an infected person coughs, sneezes, or speaks. | ** Yes ** No |
| B2 | People can become infected by touching a contaminated surface and then touching their eyes, nose, or mouth before washing their hands. | ** Yes ** No |
| B3 | Direct contact with the blood of COVID-19 patients is one way of COVID-19 transmission. | **** Yes ** No** |
|  | Other ways:  ....................................................................................................... |  |
|  | K2. COVID-19 symptoms include (B4-B11): |  |
| B4 | Fever (common symptom) | ** Yes ** No |
| B5 | Dry cough (common symptom) | ** Yes ** No |
| B6 | Tiredness (common symptom) | ** Yes ** No |
| B7 | Difficulty breathing and shortness of breath | ** Yes ** No |
| B8 | Sore throat | ** Yes ** No |
| B9 | Nausea and vomitting | ** Yes ** No |
| B10 | Headache | ** Yes ** No |
| B11 | Bellyache and diarrhea | ** Yes ** No |
|  | Other symptoms:  ....................................................................................................... |  |
|  | K3. How to prevent COVID-19 (B12-B15): |  |
| B12 | You should maintain at least one-meter distance with people coughing or sneezing | ** Yes ** No |
| B13 | Using masks, and covering your mouth and nose when coughing or sneezing are unuseful ways for COVID-19 prevention | **** Yes ** No** |
| B14 | You should wash your hands regularly with soap or alcohol-based hand rub, and not touch your face | ** Yes ** No |
| B15 | You should supplement nutrition and regularly do exercise | ** Yes ** No |
|  | Other measures:  ....................................................................................................... |  |
| **C. Attitude and practice** | | |
| C1 | Do you seek and update information on COVID-19 every day? | **** Yes **** No |
|  | Your sources of COVID-19 information include (C2-C4): |  |
| C2 | Shares from other colleagues and pharmacists | **** Yes **** No |
| C3 | The internet: social network and online newspapers | **** Yes **** No |
| C4 | Mass media (national news, radio, television) | **** Yes **** No |
|  | Other sources:  ....................................................................................................... |  |
| C5 | In the last three months, have customers asked you questions involving COVID-19? | **** Yes **** No |
|  | Common questions of customers include (C6-C9): |  |
| C6 | The ways of COVID-19 transmission | **** Yes **** No |
| C7 | Symptoms of COVID-19 | **** Yes **** No |
| C8 | COVID-19 prevention | **** Yes **** No |
| C9 | What they should do if suspecting of being infected with the COVID-19 virus | **** Yes **** No |
|  | Other questions:  .......................................................................................................  ....................................................................................................... |  |
|  | In the last three months, pharmaceutical products usually purchased in your drugstore include (C10-C13): |  |
| C10 | Vitamins and dietary supplements | **** Yes **** No |
| C11 | Medicines for treating influenza and headache | **** Yes **** No |
| C12 | Painkillers and antipyretics | **** Yes **** No |
| C13 | Antiseptics and hand sanitizers | **** Yes **** No |
|  | Other products:  .......................................................................................................  ....................................................................................................... |  |
| C14 | Do you think taking notes about the information of people who purchase drugs used to treat cough, fever and flu is necessary? | **** Yes **** No |
| C15 | Will you ask customers some information if they have symptoms the same as COVID-19 viral contamination? | **** Yes **** No |
|  | You will ask them about (C16-C19): |  |
| C16 | Their symptoms | **** Yes **** No |
| C17 | Their travel history | **** Yes **** No |
| C18 | People who they had close contact with | **** Yes **** No |
| C19 | Whether or not they update information on COVID-19 regularly | **** Yes **** No |
|  | Other questions:  .......................................................................................................  ....................................................................................................... |  |
| C20 | Have you communicated with customers when at least one person did not wear a face mask in the last three month? | **** Yes (Already)  **** No (Never) |
|  | If you already did it, list your reason(s) (C21-C24): |  |
| C21 | Because of lacking face masks in my drugstore | **** Yes **** No |
| C22 | I forgot to wear face masks | **** Yes **** No |
| C23 | Customers did not wear face masks | **** Yes **** No |
| C24 | Is there any measure to limit viral contamination and protect pharmacists and customers in your drugstore? | **** Yes **** No |
|  | Measures for COVID-19 prevention of your drugstore include  (C25-C33): |  |
| C25 | Install glass shields (barriers or partitions) | **** Yes **** No |
| C26 | Put hand sanitizers at the doors of the drugstore and require customers to use them before entering the drugstore | **** Yes **** No |
| C27 | Maintain at least one-meter distance between staff-customer | **** Yes **** No |
| C28 | Maintain at least one-meter distance between staff-staff | **** Yes **** No |
| C29 | Equip pharmacists face masks and hand sanitizers | **** Yes **** No |
| C30 | Reduce the number of pharmacists in drugstores | **** Yes **** No |
| C31 | Organize courses of training and sharing information on COVID-19 among pharmacists | **** Yes **** No |
| C32 | Indirectly give customers advice on drugs (through phones, messenger, social media...) | **** Yes **** No |
| C33 | Deliver medicines to customers’ doors | **** Yes **** No |
|  | Other measures:  .......................................................................................................  ....................................................................................................... |  |

Thank you for assisting us in data collection.

Best regards.

**Questionnaire in Vietnamese**

| **TRƯỜNG ĐẠI HỌC DƯỢC HÀ NỘI** | Mã phiếu: .....................................  Ngày phỏng vấn: ...................................... |
| --- | --- |

**PHIẾU KHẢO SÁT KIẾN THỨC, THÁI ĐỘ, THỰC HÀNH (KAP)**

**CỦA CÁC DƯỢC SĨ VỀ ĐẠI DỊCH COVID-19 TẠI VIỆT NAM**

Xin chào các anh/chị. Nhóm nghiên cứu của Trường Đại học Dược Hà Nội đang tiến hành một cuộc khảo sát về kiến thức, thái độ và thực hành của các dược sĩ về đại dịch COVID-19 tại Việt Nam. Mục tiêu của nghiên cứu là để đánh giá vai trò của những dược sĩ cộng đồng đang làm việc tại các cửa hàng thuốc trong hoạt động phòng, chống đại dịch COVID-19 tại Việt Nam. Sự đóng góp và tham gia của anh/chị sẽ góp phần quan trọng trong công cuộc đẩy lùi dịch bệnh tại Việt Nam. Thời gian trả lời câu hỏi là khoảng 10-15 phút. Mọi thông tin do anh/chị cung cấp sẽ được bảo mật theo đúng quy định và các thông tin được sử dụng chỉ cho mục đích nghiên cứu. Rất mong sự hợp tác của các anh/chị.

| **TT** | **Câu hỏi** | **Câu trả lời** |
| --- | --- | --- |
| **A. Thông tin các nhân** | | |
| A1. | Giới tính |  Nam  Nữ |
| A2. | Năm sinh | .................................. |
| A3. | Trình độ học vấn |  Đại học, thạc sĩ...   Cao đẳng   Trung cấp |
| A4. | Địa chỉ nhà thuốc | Tỉnh: ........................ |
| A5. | Kinh nghiệm bán thuốc | ....................... (năm) |
| **B. Các câu hỏi về kiến thức** | | |
|  | K1. Các cách lây truyền vi rút COVID-19 (B1-B3): |  |
| B1 | Vi rút có thể được lây truyền thông qua các hạt nhỏ được tạo ra khi một người nhiễm vi rút ho, hắt hơi hay nói chuyện. |  Đúng  Sai |
| B2 | Một người có thể bị nhiễm vi rút khi họ chạm tay vào một bề mặt đã có vi rút, không rửa tay và chạm tay vào mắt, mũi hay miệng. |  Đúng  Sai |
| B3 | Tiếp xúc trực tiếp với máu của bệnh nhân bị nhiễm vi rút có thể là con đường lây truyền vi rút COVID-19. |  Đúng  Sai |
|  | Các con đường lây truyền khác:  ............................................................................................................... |  |
|  | K2. Các triệu chứng của COVID-19 bao gồm (B4-B11): |  |
| B4 | Sốt (triệu chứng thông thường) |  Đúng  Sai |
| B5 | Ho khan (triệu chứng thông thường) |  Đúng  Sai |
| B6 | Mệt mỏi (triệu chứng thông thường) |  Đúng  Sai |
| B7 | Khó thở, thở gấp |  Đúng  Sai |
| B8 | Đau họng |  Đúng  Sai |
| B9 | Buồn nôn, nôn mửa |  Đúng  Sai |
| B10 | Đau đầu |  Đúng  Sai |
| B11 | Đau bụng, tiêu chảy |  Đúng  Sai |
|  | Các triệu chứng khác:  ............................................................................................................... |  |
|  | K3. Các biện pháp phòng ngừa sự lây lan của COVID-19 (B12-B15): |  |
| B12 | Bạn nên hạn chế tiếp xúc, duy trì khoảng cách ít nhất một mét với những người đang ho, hắt hơi |  Đúng  Sai |
| B13 | Đeo khẩu trang và che miệng, mũi khi ho, hắt hơi không phải là một biện pháp hiệu quả để phòng ngừa COVID-19 |  Đúng  Sai |
| B14 | Bạn nên rửa tay thường xuyên với xà phòng hay dung dịch rửa tay chứa cồn và hạn chế chạm vào mặt |  Đúng  Sai |
| B15 | Bạn nên bổ sung các chất dinh dưỡng và thường xuyên tập thể dục |  Đúng  Sai |
|  | Các biện pháp phòng ngừa khác:  ............................................................................................................... |  |
| **C. Thái độ và thực hành** | | |
| C1 | Bạn có tìm kiếm và truy cập thông tin về COVID-19 hàng ngày không? |  Có  Không |
|  | Nếu có, nguồn thông tin đó đến từ (C2-C4): |  |
| C2 | Chia sẻ của các đồng nghiệp, các dược sĩ khác |  Có  Không |
| C3 | Mạng internet: mạng xã hội và báo điện tử |  Có  Không |
| C4 | Các phương tiện truyền thông đại chúng (bản tin thời sự, đài, ti vi) |  Có  Không |
|  | Các nguồn khác:  ............................................................................................................... |  |
| C5 | Trong ba tháng vừa qua, khách hàng có hỏi bạn câu hỏi nào liên quan đến COVID-19 hay không? |  Có  Không |
|  | Một số câu hỏi thường gặp của khách hàng là (C6-C9): |  |
| C6 | Các con đường lây truyền COVID-19 |  Có  Không |
| C7 | Các triệu chứng khi nhiễm COVID-19 |  Có  Không |
| C8 | Các biện pháp phòng ngừa COVID-19 |  Có  Không |
| C9 | Họ nên làm gì khi nghi ngờ mình bị nhiễm COVID-19 |  Có  Không |
|  | Một số câu hỏi khác:  ...............................................................................................................  ............................................................................................................... |  |
|  | Trong ba tháng vừa qua, các loại dược phẩm mà khách hàng thường mua tại cửa hàng thuốc của bạn bao gồm (C10-C13): |  |
| C10 | Vitamin, thực phẩm bảo vệ sức khoẻ, tăng sức đề kháng |  Có  Không |
| C11 | Các thuốc điều trị cảm cúm, nhức đầu |  Có  Không |
| C12 | Thuốc giảm đau, hạ sốt |  Có  Không |
| C13 | Dung dịch rửa tay, thuốc sát khuẩn |  Có  Không |
|  | Một số sản phẩm khác:  ...............................................................................................................  ............................................................................................................... |  |
| C14 | Bạn có nghĩ là việc ghi chép lại thông tin của những khách hàng đã mua thuốc điều trị ho, sốt và cảm cúm là cần thiết không? |  Có  Không |
| C15 | Nếu khách hàng có những triệu chứng giống với triệu chứng của nhiễm COVID-19, bạn có hỏi họ thông tin gì không? |  Có  Không |
|  | Bạn sẽ hỏi họ về (C16-C19): |  |
| C16 | Các triệu chứng |  Đúng  Sai |
| C17 | Lịch sử đi lại |  Đúng  Sai |
| C18 | Những người họ từng tiếp xúc gần |  Đúng  Sai |
| C19 | Họ có tìm kiếm các thông tin về COVID-19 hàng ngày không |  Đúng  Sai |
|  | Các câu hỏi khác:  ...............................................................................................................  ............................................................................................................... |  |
| C20 | Trong ba tháng vừa qua, bạn đã bao giờ giao tiếp với khách hàng khi ít nhất có một người không đeo khẩu trang chưa? |  Đã từng   Chưa bao giờ |
|  | Nếu đã từng, các lí do bao gồm (C21-C23): |  |
| C21 | Thiếu khẩu trang |  Đúng  Sai |
| C22 | Quên không đeo khẩu trang |  Đúng  Sai |
| C23 | Khách hàng không đeo khẩu trang |  Đúng  Sai |
| C24 | Ở cửa hàng thuốc của bạn, có biện pháp nào được áp dụng để hạn chế sự lây nhiễm và bảo vệ người mua và người bán thuốc không? |  Có  Không |
|  | Các biện pháp ở cửa hàng thuốc của bạn bao gồm (C25-C33): |  |
| C25 | Dựng các tấm chắn ngăn cách (chỗ đứng tư vấn cho khách hàng) |  Có  Không |
| C26 | Đặt các chai nước rửa tay ở cửa ra vào và yêu cầu khách hàng sử dụng trước khi đi vào cửa hàng |  Có  Không |
| C27 | Duy trì khoảng cách tối thiểu 1 mét giữa người mua và người bán |  Có  Không |
| C28 | Duy trì khoảng cách tối thiểu 1 mét giữa các dược sĩ trong cửa hàng |  Có  Không |
| C29 | Trang bị khẩu trang và dung dịch rửa tay sát khuẩn cho các dược sĩ |  Có  Không |
| C30 | Giảm số lượng dược sĩ đứng bán trong cửa hàng thuốc |  Có  Không |
| C31 | Tập huấn, phổ biến và chia sẻ kiến thức về dịch bệnh giữa các dược sĩ |  Có  Không |
| C32 | Tư vấn cho khách hàng gián tiếp qua điện thoại, tin nhắn, mạng xã hội |  Có  Không |
| C33 | Triển khai dịch vụ giao thuốc tận nhà cho khách hàng |  Có  Không |
|  | Các biện pháp khác:  ...............................................................................................................  ............................................................................................................... |  |

Cảm ơn sự giúp đỡ của anh chị.

Trân trọng!
