# Supplementary material for: Knowledge, attitude and practices of community pharmacists regarding COVID-19: A paper-based survey in Vietnam
Source: PLoS One. 2021 Jul 29;16(7):e0255420. doi: 10.1371/journal.pone.0255420 (PMC8321352; doi:10.1371/journal.pone.0255420)
Supplement: Attachment — Submitted filename: PONE-D-21-08000_reviewer_RA.pdf [file pone.0255420.s003.pdf]

PLOS ONE

Knowledge, attitude and practices of community pharmacists regarding COVID-19: a paper-based survey in Vietnam

--Manuscript Draft--

**Manuscript Number:**	PONE-D-21-08000
**Article Type:**	Research Article
**Full Title:**	Knowledge, attitude and practices of community pharmacists regarding COVID-19: a paper-based survey in Vietnam
**Short Title:**	KAP of Vietnamese pharmacists about COVID-19
**Corresponding Author:**	Dai Xuan Dinh, MS<br>Hanoi University of Pharmacy<br>Hanoi, Hanoi VIET NAM
**Keywords:**	COVID-19; pharmacist; knowledge; attitude; practice; Vietnam
**Abstract:**	A descriptive cross-sectional survey was conducted using a questionnaire to interview 1,023 Vietnamese pharmacists from June to August 2020 and using R software version 4.0.3 for data analysis. The results show that pharmacists' knowledge about COVID-19 transmission, symptoms and prevention was good. However, 13.4% of them didn't know about indirect ways of viral transmission. Among three education-level groups, assistant pharmacists had the highest mark of COVID-19 knowledge. Vietnamese pharmacists daily sought and updated information on the COVID-19 pandemic through mass media, national news and the internet. 92.5% of pharmacists thought that it is necessary to make medical declarations for customers purchasing medicines to treat flu, fever and cough. In medicine outlets, there were several useful measures used to protect staff and customers such as staff were equipped with face masks and hand sanitizers, using glass shields and maintaining at least one meter distance between two people. Approximately 48% of pharmacists conceded that they communicated with customers when at least one person did not wear a face mask in the last three months. In conclusion, pharmacists should comprehend ways of COVID-19 transmission and related symptoms to be able to give customers accurate advice and restrict forgetting to wear face masks in communication with medicine purchasers.
**Order of Authors:**	Huong Thi Thanh Nguyen
	Dai Xuan Dinh, MS
	Van Minh Nguyen
**Additional Information:**	
**Question**	**Response**
**Financial Disclosure**<br><br>Enter a financial disclosure statement that describes the sources of funding for the work included in this submission. Review the submission guidelines for detailed requirements. View published research articles from *PLOS ONE* for specific examples.<br><br>This statement is required for submission and **will appear in the published article** if the submission is accepted. Please make sure it is accurate.	The author(s) received no specific funding for this work.

Powered by Editorial Manager® and ProduXion Manager® from Aries Systems Corporation

**Unfunded studies**<br>Enter: *The author(s) received no specific funding for this work.*<br><br>**Funded studies**<br>Enter a statement with the following details:<br>• Initials of the authors who received each award<br>• Grant numbers awarded to each author<br>• The full name of each funder<br>• URL of each funder website<br>• Did the sponsors or funders play any role in the study design, data collection and analysis, decision to publish, or preparation of the manuscript?<br>• **NO** - Include this sentence at the end of your statement: *The funders had no role in study design, data collection and analysis, decision to publish, or preparation of the manuscript.*<br>• **YES** - Specify the role(s) played.<br><br>* typeset	
**Competing Interests**<br><br>Use the instructions below to enter a competing interest statement for this submission. On behalf of all authors, disclose any competing interests that could be perceived to bias this work—acknowledging all financial support and any other relevant financial or non-financial competing interests.<br><br>This statement is required for submission and **will appear in the published article** if the submission is accepted. Please make sure it is accurate and that any funding sources listed in your Funding Information later in the submission form are also declared in your Financial Disclosure statement.<br><br>View published research articles from *PLOS ONE* for specific examples.	The authors have declared that no competing interests exist.

**NO authors have competing interests**<br><br>Enter: *The authors have declared that no competing interests exist.*<br><br>**Authors with competing interests**<br><br>Enter competing interest details beginning with this statement:<br><br>*I have read the journal's policy and the authors of this manuscript have the following competing interests: [insert competing interests here]*<br><br>* typeset	
**Ethics Statement**<br><br>Enter an ethics statement for this submission. This statement is required if the study involved:<br><br>• Human participants<br>• Human specimens or tissue<br>• Vertebrate animals or cephalopods<br>• Vertebrate embryos or tissues<br>• Field research<br><br>Write "N/A" if the submission does not require an ethics statement.<br><br>General guidance is provided below. Consult the submission guidelines for detailed instructions. **Make sure that all information entered here is included in the Methods section of the manuscript.**	The study was approved by the ethical committee of Hanoi University of Pharmacy, Hanoi, Vietnam (reference number 3-20/PCT-HĐĐĐ).

Powered by Editorial Manager® and ProduXion Manager® from Aries Systems Corporation

**Format for specific study types**<br><br>**Human Subject Research (involving human participants and/or tissue)**<br>• Give the name of the institutional review board or ethics committee that approved the study<br>• Include the approval number and/or a statement indicating approval of this research<br>• Indicate the form of consent obtained (written/oral) or the reason that consent was not obtained (e.g. the data were analyzed anonymously)<br><br>**Animal Research (involving vertebrate animals, embryos or tissues)**<br>• Provide the name of the Institutional Animal Care and Use Committee (IACUC) or other relevant ethics board that reviewed the study protocol, and indicate whether they approved this research or granted a formal waiver of ethical approval<br>• Include an approval number if one was obtained<br>• If the study involved *non-human primates*, add *additional details* about animal welfare and steps taken to ameliorate suffering<br>• If anesthesia, euthanasia, or any kind of animal sacrifice is part of the study, include briefly which substances and/or methods were applied<br><br>**Field Research**<br><br>Include the following details if this study involves the collection of plant, animal, or other materials from a natural setting:<br>• Field permit number<br>• Name of the institution or relevant body that granted permission	
**Data Availability**<br><br>Authors are required to make all data underlying the findings described fully available, without restriction, and from the time of publication. PLOS allows rare exceptions to address legal and ethical concerns. See the PLOS Data Policy and FAQ for detailed information.	Yes - all data are fully available without restriction

A Data Availability Statement describing where the data can be found is required at submission. Your answers to this question constitute the Data Availability Statement and **will be published in the article**, if accepted.<br><br>**Important:** Stating 'data available on request from the author' is not sufficient. If your data are only available upon request, select 'No' for the first question and explain your exceptional situation in the text box.<br><br>Do the authors confirm that all data underlying the findings described in their manuscript are fully available without restriction?	
**Describe where the data may be found in full sentences. If you are copying our sample text, replace any instances of XXX with the appropriate details.**<br><br>• If the data are **held or will be held in a public repository**, include URLs, accession numbers or DOIs. If this information will only be available after acceptance, indicate this by ticking the box below. For example: *All XXX files are available from the XXX database (accession number(s) XXX, XXX.).*<br>• If the data are all contained **within the manuscript and/or Supporting Information files**, enter the following: *All relevant data are within the manuscript and its Supporting Information files.*<br>• If neither of these applies but you are able to provide **details of access elsewhere**, with or without limitations, please do so. For example:<br><br>*Data cannot be shared publicly because of [XXX]. Data are available from the XXX Institutional Data Access / Ethics Committee (contact via XXX) for researchers who meet the criteria for access to confidential data.*<br><br>*The data underlying the results presented in the study are available from (include the name of the third party*	All relevant data are within the manuscript and its Supporting Information files.

and contact information or URL).<br>• This text is appropriate if the data are owned by a third party and authors do not have permission to share the data.<br><br>* typeset	
Additional data availability information:	

Manuscript
Click here to access/download;Manuscript;Manuscript.docx

Title page

Knowledge, attitude and practices of community pharmacists regarding COVID-19: a paper-based survey in Vietnam

Short title: KAP of Vietnamese pharmacists about COVID-19

Huong Thi Thanh Nguyen[1], Dai Xuan Dinh[1*], Van Minh Nguyen[2]

[1] Department of Pharmaceutical Management and Economics, Hanoi University of Pharmacy, Hanoi city, Vietnam.

[2] Center for Population Health Sciences, Hanoi University of Public Health, Hanoi city, Vietnam.

* Corresponding author

E-mail: dinhxuandai.224@gmail.com (DXD)

Abstract

A descriptive cross-sectional survey was conducted using a questionnaire to interview 1,023 Vietnamese pharmacists from June to August 2020 and using R software version 4.0.3 for data analysis. The results show that pharmacists' knowledge about COVID-19 transmission, symptoms and prevention was good. However, 13.4% of them didn't know about indirect ways of viral transmission. Among three education-level groups, assistant pharmacists had the highest mark of COVID-19 knowledge. Vietnamese pharmacists daily sought and updated information on the COVID-19 pandemic through mass media, national news and the internet. 92.5% of pharmacists thought that it is necessary to make medical declarations for customers purchasing medicines to treat flu, fever and cough. In medicine outlets, there were several useful measures used to protect staff and customers such as staff were equipped with face masks and hand sanitizers, using glass shields and maintaining at least one meter distance between two people. Approximately 48% of pharmacists conceded that they communicated with customers when at least one person did not wear a face mask in the last three months. In conclusion, pharmacists should comprehend ways of COVID-19 transmission and related symptoms to be able to give customers accurate advice and restrict forgetting to wear face masks in communication with medicine purchasers.

Keywords: COVID-19, pharmacist, knowledge, attitude, practice, Vietnam.

Introduction

COVID-19 is an infectious disease caused by a newly discovered coronavirus. In Wuhan City of China, first cases of COVID-19 contamination were detected on 31 Decemcer 2019 [1]. After that, this perilous virus was quickly spread globally and gave rise to an outbreak of international plague. Up to the day 28 February 2021, globally, there were nearly 113.5 million COVID-19 confirmed cases and 2,520,653 deaths. In the previous week, there were nearly 2.7 million new cases and 63,477 deaths reported [2]. This plague begot huge negative influences on local and global economies. Recently, many stringent measures have been implemented in many countries, such as social distancing, complete lockdowns with the aim of curbing COVID-19 spread [3-5]. At this time, at least seven kinds of vaccines have been rolled out all over the world. More than 200 additional vaccine candidates are in development and more than 60 vaccine candidates are in clinical development [6].

In Vietnam, up to the day 11 March 2021, there were only 2,529 COVID-19 confirmed cases and 35 deaths [7]. The number of reported cases and deaths in Vietnam is far lower than that in a myriad of other countries. Vietnam could attain this impressive accomplishment by virtue of abundant reasons. One of foremost rationales is that copious doctors and nurses have strongly fighted the uphill battle in the frontline. In addition, drugstores are commonly a reliable and first place for citizens to take advice about ailments so pharmacists play an important role in putting a curb on the spread of COVID-19 pandemic [8], [9]. Up to the day March 11 2021, there are only two published articles assessing COVID-19 related knowledge of health care professionals in Vietnam [10], [11]. There is no previous report of a nationwide study assessing the knowledge, attitude and practices (KAP) of pharmacists about COVID-19 in Vietnam. This study was conducted to survey the KAP of 1,023 community pharmacists regarding COVID-19 in Vietnam in 2020.

Materials and methods

Study design

This study was a descriptive cross-sectional survey conducted in Vietnam during the COVID-19 outbreak. The study was approved by the ethical committee of Hanoi University of Pharmacy, Hanoi, Vietnam (reference number 3-20/PCT-HĐĐĐ). After reviewing numerous articles in pubmed and WHO's website [12-15], the first draft of questionnaire was developed. To ensure the logic, suitability and validity, this questionnaire was employed in order to conduct a pilot research in 20 drugstores in Hanoi (on April 2020) before commencing being used to interview pharmacists all over Vietnam. All interviewees voluntarily answered the questionnaire and their informed consent was written.

The questionnaire started with a short introduction including the objectives, procedures, declaration of confidentiality and anonymity and the volunteer nature of the participants. It was designed in Vietnamese and embraced following main parts: pharmacists' profile (5 questions), knowledge (15 questions) and attitude/practices (33 questions) (S1 Questionnaire). Eligible participants were licensed pharmacists practicing in the community. 1,023 pharmacists residing in nine provinces of Vietnam were interviewed from June 01 to August 31 2020. By virtue of the complexity of COVID-19 plague, convenience sampling was used and data were collected with the aid of medical personnel from the Department of Health in each province. Paper questionnaires were sent to pharmacists in drugstores. After completed, they were given back to the research team by post.

Data analysis

The data were entered and analyzed using Microsoft Excel 2020 and R version 4.0.3. The descriptive analysis was undertaken using means and percentages. Data normality was

check using Shapiro-Wilk test (p value > 0.05 indicating a normally distributed continuous variable). Differences between two groups were done using Wilcoxon rank sum test.

In Vietnam, there are three main groups of medical personnel licensed to sell medicines: Uni (pharmacists who graduated universities of pharmacy with 5 years of training), Col (assistant pharmacists who graduated colleges of pharmacy with 3 years of training) and Mid (middle pharmacists who finished 2-year-training courses). Normally, Vietnamese pharmacists with higher levels of education (Master, PhD) don’t directly sell medicines in retail outlets. To compare their knowledge among areas and level-education groups, marks for each drugstore’s staff were computed based on their knowledge on three main topics: the ways in which the COVID-19 virus could be transmitted (3 questions), related symptoms (8 questions) and ways to prevent COVID-19 (4 questions). The right answer for each knowledge question was assigned 1 point and the wrong answer was assigned 0 point. The total knowledge mark of pharmacists varied between 0 (no correct answer) to 15 (all correct answer). The mark ≥10 indicated good knowledge and <10 indicated poor knowledge. Correct answers were mainly compared to information from WHO [12], [15].

Results

Participants were mainly women and most of them were aged from 20 to 39 years old (75.8%). Their time of working in drugstores was mostly less than 20 years and nearly 70% of pharmacists had less than 10 years of experience (Table 1).

Table 1. Characteristics of study sample (n =1,023)

**No.**	**Characteristics**		**Number**	**(%)**
A1	Gender	Male	223	21.8
		Female	800	78.2
A2	Age	20 - 29	320	31.3

No.	Characteristics		Number	(%)
		30 - 39	455	44.5
		40 - 49	156	15.2
		50 - 59	67	6.5
		60 – 69	20	2.0
		≥ 70	5	0.5
A3	Education level	University & higher (Uni)	364	35.6
		College (Col)	394	38.5
		Middle (Mid)	265	25.9
A4	Working experience in drugstores (years)	< 5	367	35.9
		5 – 9	309	30.2
		10 – 19	250	24.4
		20 – 29	70	6.8
		≥ 30	27	2.6
A5	Area	Caobang	33	3.2
		Hanam	95	9.3
		Hanoi	124	12.1
		Hochiminh	150	14.7
		Namdinh	52	5.1
		Nghean	26	2.5
		Thanhhoa	173	16.9
		Tuyenquang	193	18.9
		Vinhphuc	177	17.3

Almost pharmacists knew that COVID-19 virus could be transmitted by close contact with infected people via secretions of mouth and nose (direct way) while about 13.4% of them

didn't know indirect ways of transmission (touching a contaminated surface). Some COVID-19 symptoms which abundant pharmacists perceived were fever, dry cough, tiredness, short and difficult respiration. The percentages of pharmacists who knew about less common symptoms of COVID-19 (nausea, vomitting, bellyache and diarrhea) were low. In addition, most of pharmacists comprehended main ways of COVID-19 prevention (Table 2).

Table 2. Knowledge of pharmacists about COVID-19

No.	COVID-19 knowledge	Right answers (%)
	K1. Ways of COVID-19 transmission (B1-B3):	
B1	The virus that cause COVID-19 spreads primarily through droplets generated when an infected person coughs, sneezes or speaks.	1,003 (98.0)
B2	People can become infected by touching a contaminated surface and then touching their eyes, nose or mouth before washing hands.	886 (86.6)
B3	Direct contact with blood is not one way of COVID-19 transmission.	957 (93.5)
	K2. COVID-19 symptoms include (B4-B11):	
B4	Fever (common symptom)	1,016 (99.3)
B5	Dry cough (common symptom)	1,009 (98.6)
B6	Tiredness (common symptom)	867 (84.8)
B7	Difficulty breathing and shortness of breath	975 (95.3)
B8	Sore throat	461 (45.1)
B9	Nausea and vomitting	315 (30.8%)
B10	Headache	764 (74.7%)
B11	Bellyache and diarrhea	113 (11.0%)
	K3. How to prevent COVID-19 (B12-B15):	
B12	Maintain at least 1 metre distance with people coughing/sneezing	993 (97.1)

No.	COVID-19 knowledge	Right answers (%)
B13	Use mask and cover your mouth and nose when coughing/sneezing	1,005 (98.2)
B14	Wash hands regularly with soap or alcohol-based hand rub and not touch the face	976 (95.4)
B15	Supplement nutritions and regularly do exercise	955 (93.4)

The mean knowledge mark of 1,023 pharmacists was 12.02 ± 1.64, which indicates that 93.4% of pharmacists had good knowledge about COVID-19. Among three groups of education levels, it seems that pharmacists who graduated colleges of pharmacy had the highest mark. The differences of average marks among three groups mainly sprung from the knowledge of COVID-19 symptoms (Table 3). Among areas (Figure 1), the knowledge marks of staffs were seemingly equal, except for Thanh Hoa and Tuyen Quang province. In particular, the average mark about COVID-19 knowledge of pharmacists in Hanam, Hanoi capital, Hochiminh city, Thanhhoa, Tuyenquang, Vinhphuc and other provinces was 11.60, 12.13, 12.34, 13.38, 11.02, 11.97 and 11.51, respectively.

Table 3. The COVID-19 knowledge mark of pharmacists classified by education levels

Level of education	Average mark			
	K1	K2	K3	Total
**Total (n=1,023)**	**2.78**	**5.40**	**3.84**	**12.02**
Uni (n=364)	2.92	5.39	3.91	12.23
Col (n=394)	2.93	5.62	3.90	12.45
Mid (n=265)	2.37	5.07	3.65	11.09
p value*	$p_{uni\text{-}col} > 0.05$<br>$p_{col\text{-}mid} < 0.001$<br>$p_{mid\text{-}uni} < 0.001$	$p_{uni\text{-}col} = 0.0022$<br>$p_{col\text{-}mid} < 0.001$<br>$p_{mid\text{-}uni} = 0.0012$	$p_{uni\text{-}col} > 0.05$<br>$p_{col\text{-}mid} = 0.0013$<br>$p_{mid\text{-}uni} < 0.001$	$p_{uni\text{-}col} = 0.0023$<br>$p_{col\text{-}mid} < 0.001$<br>$p_{mid\text{-}uni} < 0.001$

* p values were computed using Wilcoxon rank sum test

K1 includes 3 questions (B1-B3), K2 includes 8 questions (B4-B11) and K3 includes 4 questions (B12-B15) in S1 Questionnaire.

Fig 1. The COVID-19 knowledge marks of Vietnamese pharmacists in areas

In general, Vietnamese drugstores' staffs quotidianly sought and updated COVID-19 information from mass media, national news and the internet. Approximately 89.4% staffs in drugstores was questioned by customers about COVID-19. Their popular questions included ways of COVID-19 trasmission, symptoms and prevention. There were several kinds of pharmaceutical products which customers have purchased recently, including vitamins, functional foods, antiseptics and hand sanitizers. If customers had symptoms of viral contamination, 91.0% of pharmacists would ask them (about symptoms, travel history) to take notes. Moreover, in drug outlets, there were many ways to curb the capacity of viral contamination, such as equiping staffs face masks and hand sanitizers, using glass shields, maintaining the minimum distance between two people (Table 4).

Table 4. The attitude and practices of pharmacists about COVID-19

No.	Questions	"Yes" answers (%)
C1	Do you seek and update information on COVID-19 every day?	1,018 (99.5)
	Your sources of COVID-19 information (C2-C4):	
C2	Other colleagues/pharmacists	533 (52.1)
C3	The internet/online newspapers	882 (86.2)
C4	The national news, mass media	987 (96.5)
C5	In the last 3 months, whether or not customers have asked you about information involving COVID-19	915 (89.4)
	Common questions of customers (C6-C9):	
C6	Ways of COVID-19 transmission	788 (77.0)

No.	Questions	"Yes" answers (%)
C7	COVID-19 symptoms	830 (81.1)
C8	COVID-19 prevention	857 (83.8)
C9	What they should do if suspecting of being infected with COVID-19 virus	579 (56.6)
	In the last 3 months, pharmaceutical products usually purchased in your drugstore include (C10-C13):	
C10	Vitamins and functional foods	978 (95.6)
C11	Medicines for treating influenza and headache	420 (41.1)
C12	Painkillers and febrifuges	364 (35.6)
C13	Antiseptics and hand sanitizers	842 (82.3)
C14	Do you think doing medical declaration for people who purchase drugs to treat cough, fever and flu is necessary?	946 (92.5)
C25	Will you ask customers some information if they have symptoms the same as COVID-19 viral contamination?	931 (91.0)
	You will ask them about (C26-C29):	
C26	Their symptoms	889 (86.9)
C27	Travel history	738 (72.1)
C28	People who they had close contact with	731 (71.5)
C29	Whether or not they update information on COVID-19 regularly	752 (73.5)
C30	Have you communicated with customers when at least one person did not wear face mask in the last 3 month?	490 (47.9)
	If you already did it, list your reason(s) (C31-C33):	

No.	Questions	"Yes" answers (%)
C31	Because of lacking face mask in drugstore	148 (14.5)
C32	I forgot to wear	388 (37.9)
C33	Customers did not wear face mask	19 (1.9)
C15	Is there any ways to limit viral contamination and protect staffs and customers in your drugstore?	996 (97.4)
	Ways for COVID-19 prevention in your drugstore (C16-C24):	
C16	Install glass shields (barriers or partitions)	849 (83.0)
C17	Put hand sanitizers in doors and require customers to use before coming in	969 (94.7)
C18	Maintain at least 1 metre distance between staff-customer	872 (85.2)
C19	Maintain at least 1 metre distance between staff-staff	722 (70.6)
C20	Equip staffs face mask and hand sanitizers	972 (95.0)
C21	Reduce the number of staffs in drugstores	664 (64.9)
C22	Organize courses of training and sharing information on COVID-19 among pharmacists	550 (53.8)
C23	Indirectly give customers advice on drugs (phones, messenger, social media...)	464 (45.4)
C24	Deliver medicines to customers' doors	430 (42.0)

Discussion

Results from manifold researches show that pharmacists were and are making vital contributions to hindering the expansion of pandemics [16-20]. They can bear abundant responsibilities for being the rapid point of care tests for COVID-19, taking notes about suspected cases, ensuring the medicine quality, mitigating drug shortages, supplying reliable COVID-19 information to the communities, ensuring education and home care for citizens,

suspected patients and related family members while in self isolation [21]. In China, pharmacists have played a key role in maintaining a stable drug supply, pharmacotherapy management of patients and disseminating medical knowledge to the public. When the outbreak had just commenced, pharmacists quickly compiled documents to introduce detailed information on therapeutic drugs for COVID-19. Hence, doctors could better master the characteristics and instructions of medications [22]. Pharmacists are the most accessible health care providers, having skills and knowledge to assume a crucial role in increasing COVID-19 diagnostic testing in the United States. The active engagement of pharmacists in COVID-19 testing can assist in addressing many gaps in testing availability and demonstrating the value that pharmacists can provide in addressing unmet health needs [23]. As a consequence, enhancing knowledge of COVID-19 for pharmacists is of paramount importance.

The average mark of COVID-19 knowledge of Vietnamese pharmacists was 12.02 (out of 15). The results from this study demonstrated that the majority of Vietnamese pharmacists have good knowledge about COVID-19 (93.4%), which was consistent with findings from studies from Pakistan (84%) [24], China (84%) [25], Iran (90%) [26] but far higher than Jordan (40%) [27], Ethiopia (53%) [28] and Iran (56%) [29]. Among areas, the average mark of pharmacists about COVID-19 knowledge in Thanhhoa province is the highest (13.38) while that in Tuyenquang province is the lowest (11.02). The average mark in other provinces was from 11.51 to 12.34. Thanhhoa is a flourishing province of Vietnam, thriving on health care, possessing many nimble and proficient authorities. Thanhhoa Department of Health usually organizes training courses for pharmacists to broaden their knowledge so pharmacists in this province found easy to attain high marks of knowledge. Meanwhile, Tuyenquang is a highland province. Abundant conditions in this province (such as material facilities, human resources, traffic), therefore, can be developed less than other provinces. These characteristics can be some main reasons explaining for the aforementioned results.

A special point is that the average mark of pharmacists who graduated colleges of pharmacy (Col pharmacists) was higher than that of pharmacists who graduated universities of pharmacy (Uni pharmacists) ($p < 0.05$). The time of studying and training for these two education-level groups was 3 and 5 years, respectively. The important rationale behind this fact is that colleges of pharmacy in Vietnam usually focus on instructing students in practical knowledge for opening drugstores, selling medications and these students usually work in drugstores for a long period of time. In drug outlets, Uni pharmcists are commonly the owners whereas Col and Mid pharmacists are recruited as employees. After graduation, Uni pharmacists usually work in hospitals, pharmaceutical conglomerates, not directly sell medications to patients so it seems that they don't care much about issues in the community in comparison with Col pharmacists.

COVID-19 drew Vietnamese pharmacists' attention when 98.8% of pharmacists said that they sought and updated information on COVID-19 on a daily basis. COVID-19 information was obtained from mass media, national news and the internet; being similar to results of a study in Jordan: the sources of information for pharmacists mostly came from general media, WHO reports, published research papers and Facebook [30]. In Ethiopia, internet/social media and mainstream media were also identified as sources of COVID-19 information by almost all the interviewees [28].

Roughly 89% of pharmacists told that their customers asked them about topics involving COVID-19 (including symptoms, ways of viral transmission and how to prevent viral contamination). Thank to good knowledge on COVID-19, Vietnamese pharmacists can find easy to give customers useful advice. In addition, 92.5% of pharmacists thought that it is necessary to take notes about customers purchasing medicines to treat cough, fever and flu. 91% of pharmacists would ask customers their information (symsptoms, travel history) if they have symptoms the same as COVID-19 viral contamination. These beneficial activities played

a not inconsiderable part in bringing about impressive achievements in the battle against COVID-19 pandemic in Vietnam.

During the period of COVID-19 outbreak, drugstores' employees were equiped with face masks and hand sanitizers. In many drugstores, glass shields were installed in order to restrict the spread of droplets from saliva/nose when the face-to-face communication between staffs and customers occurs. Hand sanitizers were also put in the doors of drugstores and purchasers were encouraged to use them before entering the drugstores. These useful activities contributed to protecting both staffs and customers. However, 47.9% of pharmacists conceded that they communicated with costumers when there was at least one person not wearing face masks in the last three months. The main reason is that staffs forgot to use face mask. There is no denying that continual wearing face mask in many hours can render anyone uncomfortable. When drugstores had no customer, staffs usually took off the face masks. They can forget to use them when customers came into. In case of forgeting to wear face masks, staffs could be protected since 86.3% of drugstores had fixed glass shields which can assist in reducing the capacity of viral contamination.

To prevent the transmission of COVID-19 virus, Vietnam had a multitude of useful and practical remedies, such as isolating infected individuals, tracing and quarantining their contacts. From April to June 2020, schools were closed and activities for large crowds (like festivals, conferences) were cancelled. Citizens were encouraged to stay at home to minimize exposure and viral transmission. More importantly, face masks and hand sanitizers has been highly encouraged to be used. To hinder the capacity of spread from other countries, several rigorous measures were imposed, including a temporary suspension of entry of all foreigners who have come from or transited through COVID-19 affected areas and a new mandatory regulation that all incoming travellers to Vietnam have to be quarantined at centralized facilities for 14 days [31], [32]. With good knowledge about COVID-19, Vietnamese

pharmacists bears numerous responsibilities and make a not inconsiderable contribution in the battle against COVID-19 pandemic along with doctors and nurses.

Limitations

This national survey is the first paper-based study conducted to investigate the KAP of pharmacists about the COVID-19 pandemic in Vietnam with a large sample size. Many useful measures against the spread of this virus mentioned in this article can be used and applied in other countries. Most of recent KAP studies involving COVID-19 were online survey. A paper-based study has several following strengths: generate much higher response rates than an online survey, easily and suitably approach various kinds of respondents who are not technology savvy (like old people).

However, this research has some limitations. Besides the lack of human resources, there were difficulties in data collection. In fact, social distancing was employed two times in Vietnam in April and July of 2020 because of the discovery of many COVID-19 patients in metropolises. Hence, the time for data collection was quite long and in some provinces, the number of surveyed pharmacists was low. Furthermore, questions about COVID-19 knowledge only focused on three topics: ways of COVID-19 transmission, symptoms and prevention. The results may not reflect the comprehensive knowledge of pharmacists about this pandemic.

Conclusions

The knowledge, attitude and practices of Vietnamese pharmacists about COVID-19 were good. In drugstores, there are many ways to curb viral contamination and protect staffs such as using glass shields, equiping staffs with hand sanitizers and face masks. However, Vietnamese pharmacists should comprehend ways of COVID-19 transmission and related

symptoms to be able to give customer accurate advice and restrict forgetting wearing face masks in communication with medicine purchasers.

Funding

The authors received no specific funding for this work.

Competing interests

The authors declare that there is no competing interests.

Contributors

Huong TTN: Conceptualization, Methodology, Investigation, Writing – Review & Editing, Supervision, Validation. **Dai XD:** Methodology, Software, Formal analysis, Investigation, Data curation, Visualization, Writing – Original Draft Preparation, Writing – Review & Editing. **Van MN:** Conceptualization, Methodology, Investigation, Writing – Review & Editing, Project administration.

References

1. World Health Organization. Pneumonia of unknown cause – China. 2020 Jan 05 [Cited 2021 Mar 11]. Available from: https://www.who.int/csr/don/05-january-2020-pneumonia-of-unkown-cause-china/en/

2. World Health Organization. Coronavirus disease (COVID-19): Weekly epidemiological update. 2021 Mar 02 [Cited 2021 Mar 11]. Available from: https://www.who.int/publications/m/item/weekly-epidemiological-update---2-march-2021

3. Lewnard JA, Lo NC. Scientific and ethical basis for social-distancing interventions against COVID-19. *Lancet Infect Dis*. 2020; 30190-0:S1473–3099.

4. Barrot JN, Grassi B, Julien S. Sectoral effects of social distancing. 2020 Apr 02 [Cited 2021 Mar 11]. Available from:

https://ssrn.com/abstract=3569446 or http://dx.doi.org/10.2139/ssrn.3569446

5. Thunstorm L, Newbold CS, Finnoff D, Ashworth M, Shogren FJ. The benefits and costs of using social distancing to flatten the curve for COVID-19. *J Benefit Cost Anal.* 2020;2: 179-195. doi: 10.1017/bca.2020.12.

6. World Health Organization. COVID-19 vaccines. [Cited 2021 Mar 11]. Available from: https://www.who.int/emergencies/diseases/novel-coronavirus-2019/covid-19-vaccines

7. Vietnam Ministry of Health. Information on COVID-19 virus. 2021 March 11 [Cited 2021 Mar 11]. Available from: https://ncov.moh.gov.vn/

8. Khan Z, Muhammad K, Ahmed A, Rahman H. Coronavirus outbreaks: prevention and management recommendations. *Drugs Ther Perspect.* 2020; 36: 215–217. https://doi.org/10.1007/s40267-020-00717-x

9. Ung COL. Community pharmacist in public health emergencies: quick to action against the coronavirus 2019-nCoV outbreak. *Social Administrative Pharmacy*. 2020;16(4):583–586. PMID:32081569

10. Huynh G, Nguyen TN, Tran VK, Vo KN, Vo VT, Pham LA. Knowledge and attitude toward COVID-19 among healthcare workers at District 2 Hospital, Ho Chi Minh City. *Asian Pac J Trop Med*. 2020;13:260-265.

11. Nguyen TTP, Nguyen LH, Le HT, Vu GT, Hoang MT, Nguyen DN et al. Perceptions and Attitudes Toward COVID-19-Related National Response Measures of Vietnamese: Implications for Pandemic Prevention and Control. *Front. Public Health.* 2020;8:589053. doi: 10.3389/fpubh.2020.589053

12. World Health Organization. Coronavirus: Overview, symptoms, prevention. [Cited 2021 March 11]. Available from: https://www.who.int/health-topics/coronavirus#tab=tab_1

13. World Health Organization. Coronavirus disease (COVID-19) pandemic. [Cited 2021 March 11]. Available from: https://www.who.int/emergencies/diseases/novel-coronavirus-2019

14. World Health Organization. Rational use of personal protective equipment for coronavirus disease (COVID-19) and considerations during severe shortages. 2020 April 6.

15. World Health Organization. Q&As on COVID-19 and related health topics. 2020 Mar 11 [Cited 2021 Mar 11]. Available from: https://www.who.int/emergencies/diseases/novel-coronavirus-2019/question-and-answers-hub

16. Strand MA, Bratberg J, Eukel H, Hardy M, Williams C. Community Pharmacists' Contributions to Disease Management During the COVID-19 Pandemic. *Preventing chronic disease*. 2020;17,E69. https://doi.org/10.5888/pcd17.200317.

17. Australian Healthcare and Hospital Association. Improving Pharmacist Involvement in Pandemic Influenza Planning and Response in Australia. 2018 March 14 [Cited 2021 Mar 11]. Available from: https://ahha.asn.au/system/files/docs/publications/summary_deeble_institute_issues_brief_no._25.pdf

18. Gross AE, MacDougall C. Roles of the clinical pharmacist during the COVID- 19 pandemic. *Journal of the American Colledge of Clinical Pharmacy*. 2020; 1–3. https://doi.org/10.1002/jac5.1231.

19. Li H, Zheng S, Liu F, Liu W, Zhao R. Fighting against COVID-19: innovative strategies for clinical pharmacists. *Res Soc Adm Pharm.* 2020;1:1813-1818. doi: 10.1016/j.sapharm.2020.04.003.

20. Cadogan CA, Hughes CM. On the frontline against COVID-19: community pharmacists' contribution during a public health crisis. *Res Soc Adm Pharm.* 2021:1:2032-2035. doi: 10.1016/j.sapharm.2020.03.015.

21. Erku DA, Belachew SA, Abrha S, Sinnollareddy M, Thomas J, Steadmen KJ *et al*. When fear and misinformation go viral: Pharmacists' role in deterring medication misinformation during the 'infodemic' surrounding COVID-19. *Res Soc Adm Pharm.* 2021:1:1954-1963. https://doi.org/10.1016/j.sapharm.2020.04.032

22. Tan SL, Zhang BK, Xu P. Chinese pharmacists' rapid response to the COVID-19 outbreak. *AJHP*. 2020; 77: 1096–1097. https://doi.org/10.1093/ajhp/zxaa120.

23. Goode JR, Page A, Burns A, Bernard S, Wheawill S, Gatewood SBS. The pharmacist's role in SARS-CoV-2 diagnostic testing. *J Am Pharm Assoc (2003).* 2020;60(6):e19-e32. doi:10.1016/j.japh.2020.08.017

24. Hussain I, Majeed A, Saeed H, Hashmi FK, Imran I, Akbar M, et al. A national study to assess pharmacists' preparedness against COVID-19 during its rapid rise period in Pakistan. *PLoS ONE.* 2020;15(11): e0241467. https://doi.org/10.1371/journal.pone.0241467

25. Zhou M, Tang F, Wang Y, Nie H, Zhang L, et al. Knowledge, attitude and practice regarding COVID-19 among health care workers in Henan, China. *Journal Hospital Infections*. 2020;105(2):183–187. PMID:32278701.

26. Nemati M, Ebrahimi B, Nemati F. Assessment of Iranian Nurses' Knowledge and Anxiety Toward COVID-19 During the Current Outbreak in Iran, *Arch Clin Infect Dis*. 2020; 15(COVID-19):e102848. doi: 10.5812/archcid.102848.

27. Khader Y, Al Nsour M, Al-Batayneh OB, Saadeh R, Bashier H, et al. Dentists' Awareness, Perception, and Attitude Regarding COVID-19 and Infection Control: Cross-Sectional Study Among Jordanian Dentists. *JMIR Public Health and Surveillance*. 2020;6: e18798. PMID:32250959

28. Tesfaye ZT, Yismaw MB, Negash Z, Ayele AG. COVID-19-Related Knowledge, Attitude and Practice Among Hospital and Community Pharmacists in Addis Ababa, Ethiopia. *Integr Pharm Res Pract.* 2020;9:105-112. doi: 10.2147/IPRP.S261275. PMID: 32904494.

29. Gohel KH, Patel PB, Shah PM, Patel JR, Pandit N, Raut A. Knowledge and perceptions about COVID-19 among the medical and allied health science students in India: An online cross-sectional survey. Clinical Epidemiological Global Health. 2020;10.1016/j.cegh.2020.07.008. PMID:32838066

30. Basheti IA, Nassar R, Barakat M, Alqudah R, Abufarha R, Mukattash TL et al. Pharmacists' readiness to deal with the coronavirus pandemic: Assessing awareness and perception of roles. *Res Social Adm Pharm.* 2020:S1551-7411(20)30418-6. doi: 10.1016/j.sapharm.2020.04.020. Epub ahead of print. PMID: 32360181; PMCID: PMC7194707.

31. Nguyen THD, Vu DC. Summary of the COVID-19 outbreak in Vietnam - Lessons and suggestions. *Travel Med Infect Dis.* 2020;37:101651. doi: 10.1016/j.tmaid.2020.101651. Epub 2020 Apr 2. PMID: 32247928; PMCID: PMC7146658.

32. Dinh L, Dinh P, Nguyen PDM, Nguyen DHN, Hoang T. Vietnam's response to COVID-19: prompt and proactive actions. *J Travel Med.* 2020;27(3):taaa047. doi: 10.1093/jtm/taaa047. PMID: 32297929; PMCID: PMC7184391.

Supporting information

S1 Questionnaire

S2 Data file

Figure 1. The COVID-19 knowledge marks of Vietnamese pharmacists in areas

Click here to access/download;Figure;Figure 1.tiff

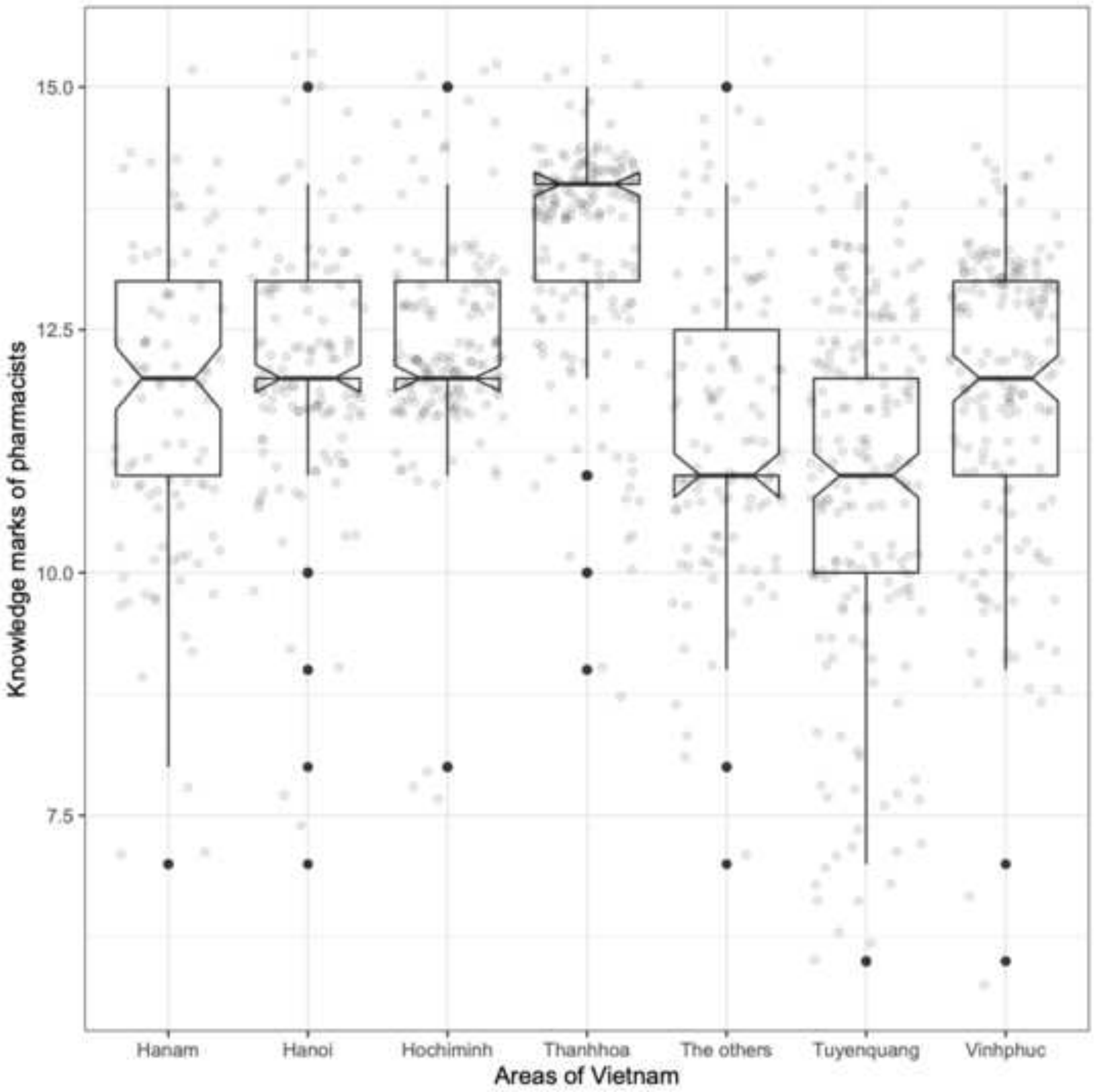

S1 Questionnaire

Click here to access/download
Supporting Information
S1 Questionnaire.docx

S2 Data file

Click here to access/download
Supporting Information
S2 Data file.xlsx
