# Supplementary material for: Knowledge, attitude and practices of community pharmacists regarding COVID-19: A paper-based survey in Vietnam
Source: PLoS One. 2021 Jul 29;16(7):e0255420. doi: 10.1371/journal.pone.0255420 (PMC8321352; doi:10.1371/journal.pone.0255420)
Supplement: Attachment — Submitted filename: Response to Reviewers.docx [file pone.0255420.s004.docx]

**REBUTTAL LETTER**

Dear editors and reviewers.

First and foremost, all authors want to express our deep gratitude to editors and reviewers who gave us valuable and practical advice. We carefully read your comments and revised our manuscript. Our responses are written in blue color. Thank you so much for your assistance.

Best regards.

**......................................................................................................**

**Journal Requirements**

When submitting your revision, we need you to address these additional requirements.

1. Please ensure that your manuscript meets PLOS ONE's style requirements, including those for file naming. The PLOS ONE style templates can be found at

https://journals.plos.org/plosone/s/file?id=wjVg/PLOSOne_formatting_sample_main_body.pdf and

https://journals.plos.org/plosone/s/file?id=ba62/PLOSOne_formatting_sample_title_authors_affiliations.pdf

We endeavored to revise mistakes in our manuscript.

2. We suggest you thoroughly copyedit your manuscript for language usage, spelling, and grammar. If you do not know anyone who can help you do this, you may wish to consider employing a professional scientific editing service.

Upon resubmission, please provide the following:

• The name of the colleague or the details of the professional service that edited your manuscript

• A copy of your manuscript showing your changes by either highlighting them or using track changes (uploaded as a *supporting information* file)

• A clean copy of the edited manuscript (uploaded as the new *manuscript* file)

We strived to correct typos and grammar.

In addition, Ms. Chi Nguyen Phuong, a Research Ph.D. Student from the University of Groningen, Netherlands, assisted us in language editing.

3. Please include additional information regarding the survey or questionnaire used in the study and ensure that you have provided sufficient details that others could replicate the analyses. For instance, if you developed a questionnaire as part of this study and it is not under a copyright more restrictive than CC-BY, please include a copy, in both the original language and English, as Supporting Information.

Moreover, please include more details on how the questionnaire was pre-tested, and whether it was validated.

The original questionnaire written in Vietnamese was added in the Supporting Information (S1 Questionnaire).

The pre-test and validation of our questionnaire were added on page 5 (lines 93 - 98).

4. In your Methods section, please provide additional information about the participant recruitment method and the demographic details of your participants.

Please ensure you have provided sufficient details to replicate the analyses such as:

a) the recruitment date range (month and year),

b) a description of any inclusion/exclusion criteria that were applied to participant recruitment,

c) a table of relevant demographic details,

d) a statement as to whether your sample can be considered representative of a larger population,

e) a description of how participants were recruited, and

f) descriptions of where participants were recruited and where the research took place.

Information on the aforementioned requirements can be found on pages 5 - 7 (lines 92 - 124).

**Review Comments to the Author**

Reviewer #1

The manuscript is an excellent topic within this time we are living in. It discusses the KAP of COVID-19 in Vietnamese pharmacist. I have added my notes to the attached PDF file, but I would like to highlight two points of mine:

1. Would the authors consider performing a regression analysis of independent variables to check which of these factors is affecting the pharmacists' knowledge the most?

We used the analysis of covariance (ANCOVA), a general multivariate model to identify factors associated with pharmacists’ knowledge scores.

The model reported in Table 4 was the best model chosen by using the Bayesian Model Averaging method (packages BMA and BAS in R software version 4.0.4).

2. Would the authors clarify the difference between different provinces and different health professions a bit further.

We added some information and explanations for these results in the Discussion section

(lines 222 - 253).

3. Could the difference in scores be due to years of experience? Regression analysis of different independent variables affecting knowledge score would be a good tool to find out.

When working experience, a continuous variable, is divided into five subgroups, there are differences in knowledge scores among subgroups: less than 5 years, 5-to-9, 10-to-14, 15-to-19, and $\geq$20 years (p < 0.001) (Table 3). However, in the multivariate linear model (Table 4), this factor was excluded because there is a strong relationship between the age and the working experience of pharmacists in medicine outlets (p < 0.001, *Spearman’s rank correlation*). We used the Bayesian Model Average method for selecting the best model (income variables include age, gender, education level, working experience, and residence). The results from this model show that the knowledge score was significantly associated with pharmacists’ age, education level, and residence (p < 0.001).

4. Line 124: Sometimes there are confusions about what is meant with "functional foods" and some might use the term "nutraceuticals”. Can the authors give a clear definition of what is meant with "functional foods" or to give examples in order to clarify.

We used “Dietary Supplements” to supersede “Functional foods”. The definition of dietary supplements was added at the end of Table 5.

In 2014, the Vietnam Ministry of Health published a Circular regulating the management of functional foods. The definition of the dietary supplement was taken from this Circular. <https://vanbanphapluat.co/circular-no-43-2014-tt-byt-regulating-the-management-of-functional-foods>

5. This study included dentists. I believe that there are Jordanian studies about pharmacists. The other referenced studies within this paragraph includes nurses and healthcare workers and not pharmacists. The comparison would be better represented if done among studies including pharmacists.

References involving healthcare workers not pharmacists were removed.

In addition, many previous studies on pharmacists were added [reference 15 - 30].

6. Results (line 99 - 104): Sentences can be clarified. Add numerical percentage for clarity.

Line 158 – 159, 179 - 184: Paraphrase the sentence to clarify.

Line 186 – 187, 211 – 212: Could "not inconsiderable" be stated as considerable. The double negatives are confusing.

We revised these mistakes.

Reviewer #2:

This paper discusses Vietnamese pharmacists’ knowledge, attitude and practices regarding the COVID-19 pandemic through administering a national paper-based survey. There were 1,023 responses that were analyzed to look at the demographics, knowledge, attitudes, and practices with patients through the studies time frame (June to August 2020). The authors analyzed the responses based on descriptive statistics to gather the results. There were major issues with the manuscript, for example:

1. There is no report on the response rate from all pharmacists who were given the survey.

This information can be found in lines 108 - 110.

2. There was a majority of female respondents and no analysis was done to show if this was a confounder.

What does the P values listed in Table 3 signify?

We used the analysis of covariance (ANCOVA), a general multivariate linear model, to identify factors associated with the knowledge scores of pharmacists. The model was chosen by using the Bayesian Model Averaging method. The old Table 3 was removed. New results can be seen in Table 3 and Table 4.

3. Authors attributed Vietnam’s unique low number of COVID cases/deaths to two reasons “copious doctors and nurses have strongly fighted the uphill battle in the frontline. In addition, drugstores are commonly a reliable and first place for citizens to take advice about ailments”. The reviewer is not sure how Vietnamese doctors and pharmacists are achieving significantly better outcomes than their global counterparts who can claim are doing the exact same measures.

We revised this mistake. We listed some possible reasons explaining why the COVID-19 confirmed cases and deaths in Vietnam were low. We did not mean to compare the roles and achievements among health workers or countries. We are truly sorry for this misunderstanding.

4. In looking into attitudes and practices of pharmacists, the survey included mainly non-reliable sources like other colleagues, internet and media, it didn’t include any scientific primary or secondary literature sources, WHO reports, even governmental health agencies. The authors called it “good knowledge” in line 181.

We added some information on pages 14, 19, and 20.

In the data collection form, for questions that we think that pharmacists can have other answers to (such as the question about sources of COVID-19 information), there are some blank rows in which pharmacists can write their additional answers.

In districts/provinces, community pharmacists are required to participate in online groups (on apps, such as Messenger and Zalo). These online groups are sites where pharmacists can share their news and experience, and health agencies can directly and quickly send accurate information on COVID-19 to all pharmacists. In addition, monthly, health agencies commonly organize meetings/courses to enhance the knowledge and practices of pharmacists.

In Vietnam, in fact, due to the language barriers, a majority of pharmacists only access sources of COVID-19 information spoken or written in Vietnamese. WHO reports and scientific literature sources spoken or written in English will be translated and propagandized through national news on television (by the government and health agencies). In addition, Facebook is extremely popular in Vietnam. The government and health agencies have some official pages on Facebook (for example https://www.facebook.com/tintucvtv24/), and accurate and reliable information can be disseminated to not only pharmacists but also the public. For online newspapers, the government has the website “vnexpress.net”. Everyone can find news and related information on COVID-19 from these sources. Last but not least, information on COVID-19 was strictly controlled by the government. For example, if a person posts inaccurate or controversial information on Facebook, he/she will be quickly fined.

Each person has their favorite sources to seek information they need. We think that people aged 18 and older (especially pharmacists) have to know how to distinguish between inaccurate and reliable information. Whether the sources were reliable or not, our results showed that 93.4% of interviewed pharmacists had good knowledge about COVID-19.

5. The authors did not elaborate in the results or discussion why there was a question in the survey about the pharmaceutical products usually purchased in drugstores.

We added some information in lines 189 - 191, 269 - 274. Vitamins and dietary supplements can be used for boosting the immune system. Antiseptics and hand sanitizers can be used for COVID-19 prevention as per the recommendations of the Vietnamese Ministry of Health.

In the period of time of COVID-19 outbreak, the fact that these products were usually purchased showed that the COVID-19 prevention knowledge of the public was fairly good and activities involving propagandizing COVID-19 prevention of the government may be effective.

6. Table 4, C14: the reviewer is not sure of the meaning (or legality) when the authors mention asking patients about “medical declaration” when they purchase cold and flu medicine.

We revised this mistake. This is a translation mistake when we wrote the first draft. The phrase “medical declaration” was superseded by “taking notes”. For this question, we only wanted to ask about the attitude of pharmacists (not the practices). Several symptoms the same as COVID-19 contamination (such as cough and sore throat) can be omitted by patients if these symptoms are mild. Taking notes about the information (address, phone number) of customers purchasing medicines treating cold and flu can assist health agencies to trace and contact people with suspected COVID-19. In fact, at present, in Hanoi, the Department of Health required all pharmacists to collect information on these customers to contact them if necessary. In addition, everyone can access and easily do their medical declarations on the website of the government when necessary (only about 3 minutes). Citizens are recommended to do their medical declarations if suspecting of being infected with the COVID-19 virus.

7. Line 150, 151 mentions statistics about Iran with two different percentages.

Line 155: What do authors mean by “possessing many nimble and proficient authorities”?

We revised this mistake.

8. Line 170-171: The authors did not offer adequate explanation for their results on why assistant pharmacists with 3 years of training scored higher in COVID-19 knowledge than “Uni” with 5 years training, mentioning that “it seems that they (Unis) don’t care much about issues in the community in comparison with Col pharmacists”!

The explanation for our results on education level can be seen in lines 222 - 239.

9. The reviewer thinks the statement in line 220 on how paper surveys “approach various kinds of respondents who are not technology savvy (like old people)” is considered ageism and should be modified or removed.

We revised this mistake and removed the example “old people”.
