# Supplementary material for: Knowledge, attitude and practices of community pharmacists regarding COVID-19: A paper-based survey in Vietnam
Source: PLoS One. 2021 Jul 29;16(7):e0255420. doi: 10.1371/journal.pone.0255420 (PMC8321352; doi:10.1371/journal.pone.0255420)
Supplement: Attachment — Submitted filename: Response to Reviewers.docx [file pone.0255420.s005.docx]

**REBUTTAL LETTER**

Dear editors and reviewers.

First and foremost, we want to express our deep gratitude to editors and reviewers who gave us valuable and practical advice. We carefully read your comments and revised our manuscript. Our responses are written in blue color. Thank you so much for your assistance.

Best regards.

**......................................................................................................**

**Journal Requirements**

Please review your reference list to ensure that it is complete and correct. If you have cited papers that have been retracted, please include the rationale for doing so in the manuscript text, or remove these references and replace them with relevant current references. Any changes to the reference list should be mentioned in the rebuttal letter that accompanies your revised manuscript. If you need to cite a retracted article, indicate the article’s retracted status in the References list and also include a citation and full reference for the retraction notice.

The first reference (from WHO) was removed and another article with similar information was cited.

Regarding the second, the third, and the 6th references, some new information involving COVID-19 confirmed cases, deaths (in Vietnam and global), and COVID-19 vaccines was updated in the revised manuscript.

We checked twice whether or not articles were retracted (in Pubmed and Google Scholar). If any reference should be removed, please let us know.

**Review Comments to the Author**

Reviewer #2:

1. The statement "In addition, a majority of Uni pharmacists who directly sell medicines in medicine outlets are graduates from private universities of pharmacy" is not supported by any reference. This information is also not captured in the survey and cannot be verified. Accordingly the explanation in lines 232-237 should be removed.

Although that is the reality in our country, we do not have any references to cite. Some information in lines 232-237 is quite sensitive. Therefore, we removed these lines. Thank reviewer #2 for this practical advice.

2. Table 3 in the changes tracked version contain the name of the provinces which matches the data analysis. This got dropped in the cleaned up version of the paper! Please make sure the final version has the breakdown of the region by the provinces.

We added the names of provinces for each region in Table 3. In Table 3, we did not analyze the COVID-19 knowledge scores of pharmacists in each province because they were presented in Figure 1 with box plots and lines 213-220.
